# Mechanism of sorafenib resistance associated with ferroptosis in HCC

**DOI:** 10.3389/fphar.2023.1207496

**Published:** 2023-06-07

**Authors:** Lingling Guo, Cuntao Hu, Mengwen Yao, Guang Han

**Affiliations:** Department of Anesthesiology, Shengjing Hospital of China Medical University, Shenyang, China

**Keywords:** hepatocellular carcinoma, ferroptosis, sorafenib, sorafenib resistance, ROS

## Abstract

Hepatocellular carcinoma (HCC) is the most familiar primary hepatic malignancy with a poor prognosis. The incidence of HCC and the associated deaths have risen in recent decades. Sorafenib is the first drug to be approved by the Food and Drug Administration (FDA) for routine use in the first-line therapy of patients with advanced HCC. However, only about 30% of patients with HCC will be benefited from sorafenib therapy, and drug resistance typically develops within 6 months. In recent years, the mechanisms of resistance to sorafenib have gained the attention of a growing number of researchers. A promising field of current studies is ferroptosis, which is a novel form of cell death differing from apoptosis, necroptosis, and autophagy. This process is dependent on the accumulation of intracellular iron and reactive oxygen species (ROS). Furthermore, the increase in intracellular iron levels and ROS can be significantly observed in cells resistant to sorafenib. This article reviews the mechanisms of resistance to sorafenib that are related to ferroptosis, evaluates the relationship between ferroptosis and sorafenib resistance, and explores new therapeutic approaches capable of reversing sorafenib resistance in HCC through the modulation of ferroptosis.

## Graphical Abstract

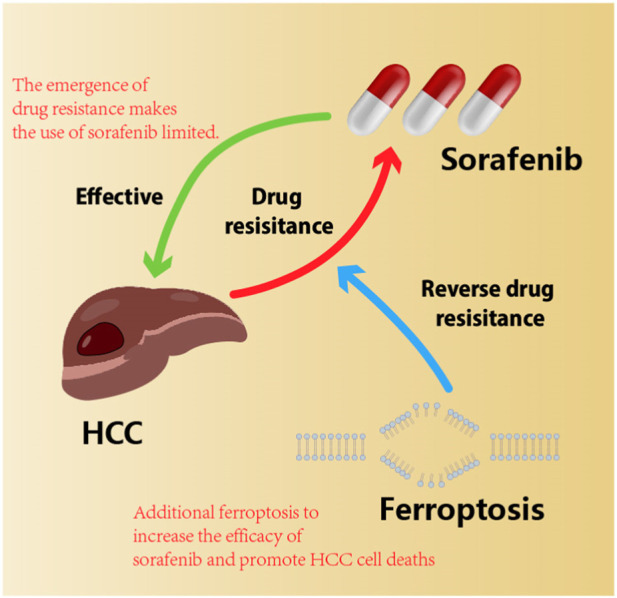

## 1 Introduction

Ferroptosis is a novel form of cell death different from apoptosis, necrosis, and autophagy (Dixon et al., 2012), the occurrence of which is dependent on the accumulation of both intracellular iron and ROS (Latunde and Gladys, 2017). The significant features of ferroptosis are cytological changes that include the reduction or loss of mitochondrial cristae, disruption of the outer mitochondrial membrane, and condensation of the mitochondrial membrane (Xie et al., 2016).

Primary liver cancer is the sixth most common cancer worldwide and the third most common cause of cancer-related death in 2020 (Sung et al., 2021). The prevalence of this disease is increasing worldwide. In addition, it is predicted that by 2025, one million people will be diagnosed with liver cancer each year (Renne et al., 2021). Primary liver cancer is divided into three subtypes: intrahepatic cholangiocarcinoma (iCCA), hepatocellular carcinoma (HCC), and other infrequent tumors, such as hepatoblastoma and carcinoma (Sia et al., 2017). HCC is responsible for between 85% and 90% of primary liver cancers worldwide (El-Serag and Lenhard Rudolph, 2007) and carries a dismal prognosis. Infections [such as chronic hepatitis B virus (HBV) and hepatitis C virus (HCV), and hepatic schistosomiasis in endemic areas], behavioral factors (alcohol and tobacco), metabolic factors (excessive body fat), and aflatoxins are among the major risk factors for the development of primary liver cancer (Chuang, Vecchia, and Boffetta, 2009). Recognition of the epidemiology, risk factors, molecular profiles, and clinical relevance of HCC has led to momentous advances in prevention, early detection, diagnosis, and therapy (Yang et al., 2019).

Changes in mitochondrial metabolism, such as the mitochondrial stress response, metabolic reprogramming, and mitochondrial proteasomal abnormalities, are highly correlated with the development and metastasis of HCC (Lee et al., 2021). The major role of mitochondria is to deliver energy to cells through oxidative phosphorylation (OXPHOS) (Wang et al., 2020). Defects in mitochondria will also result in OXPHOS damage, mitochondrial dysfunction, and increased ROS production. Given that an important cytological hallmark of ferroptosis is altered mitochondrial morphology (Xie et al., 2016), while ROS is also a major product of the ferroptosis process (Li X et al., 2020), it has been hypothesized that ferroptosis also has a significant effect on the formation of HCC. Two key molecules of the ferroptosis pathway are the cystine/glutamate antiporter (xCT) system (Liu, Zhu, and Pei, 2021) and glutathione peroxidase 4 (GPX4) (Seibt, Proneth, and Conrad, 2019). Inhibition of the xCT system and GPX4 may be beneficial in clearing cancer cells that are resistant to conventional chemotherapy or radiation therapy (Shin et al., 2018). Thus, it is important to illustrate the relationship between ferroptosis and resistance to sorafenib in HCC patients.

## 2 Relationship between HCC and sorafenib resistance

### 2.1 Rapid evolution of tumors and limitations of sorafenib’s action

The therapeutic approach to HCC depends on the stage of the disease, presenting condition and comorbidities of the patients, degree of the existing expertise developed in hepatic functional reserve, and degree of portal hypertension (Yegin et al., 2016). HCC can be treated with surgical resection, liver-directed therapy, systemic therapy, and liver transplantation (Kim, Talati, and Kim, 2017). While early stage HCC is usually resectable, advanced-stage HCC usually requires systemic therapy with sorafenib following local therapies such as ablation, transarterial chemoembolization, or external beam irradiation (Cheng, Wei-Qi, and Jin, 2020). Surgical treatment such as radical hepatectomy remains the first-line treatment choice for patients with HCC in routine clinical practice (Liu and Song, 2021). However, because of the high rates of postoperative recurrence and metastasis in them, the overall survival (OS) of patients after HCC resection remains weak (Zhang W et al., 2021). As indications for surgery for HCC expand and the likelihood of postoperative recurrence increases, research hotspots are gradually shifted toward postoperative adjuvant treatment with the aim of achieving higher resectability rates and lower recurrence rates (Akateh et al., 2019). Neoadjuvant therapy will emerge as a promising new field of research for the therapy of resectable HCCs that have a high risk of recurrence.

Sorafenib, also known as Nexavar, is a multi-targeted tyrosine kinase inhibitor (TKI) (Llovet et al., 2018) that inhibits tumor cell proliferation by inhibiting kinase activity in the B-Raf, Raf-1, and Ras/Raf/MEK/ERK signaling pathways (Wilhelm et al., 2004) and can also inhibit angiogenesis (Chang et al., 2007), as well as promote apoptosis of tumor cells. It was approved by the FDA in 2006 for the management of late-stage renal cell carcinoma and in 2007 as the only targeted drug for the therapy of advanced-stage HCC (Zhu et al., 2017). Sorafenib can lengthen the median survival time of HCC patients. In the randomized, double-blind, multicenter, phase III Sorafenib HCC Assessment Randomized Protocol (SHARP) trial (*n* = 602), the median duration of survival was 7.9 months with placebo and 10.7 months with sorafenib (Simpson and Keating, 2008). It is also known to be related to severe adverse side effects and often drug resistance within 6 months (Tian et al., 2022), suggesting both primary and acquired resistance to sorafenib in HCC cells (Chen et al., 2015). Thus, for HCC, the current use of sorafenib is somewhat a limitation. Elucidating the molecular basis of resistance to sorafenib may help improve this chemotherapeutic drug’s efficacy.

### 2.2 Exploration of sorafenib resistance pathways associated with ferroptosis

#### 2.2.1 Hippo-YAP signaling axis

The Hippo pathway, a critical regulator of tissue growth, controls transcription programs and is defined as the regulation of cell proliferation, differentiation, and migration in developing organs (Ma et al., 2019). Dysregulation of the Hippo pathway results in abnormal cell growth and tumor formation (Meng, Moroishi, and Guan, 2016). YAP/TAZ are transcriptional effectors of the Hippo signaling pathway that can promote tissue growth, regulate cell viability, and participate in a variety of physiopathological processes by regulating the activity of TEADs and SMADs (Hong and Guan, 2012). In most solid tumors, YAP/TAZ is critical for tumor initiation or growth. YAP/TAZ activation can induce cancer stem cell origin, proliferation, chemoresistance, and metastasis (Zanconato, Cordenonsi, and Piccolo, 2016).

**FIGURE 1:**
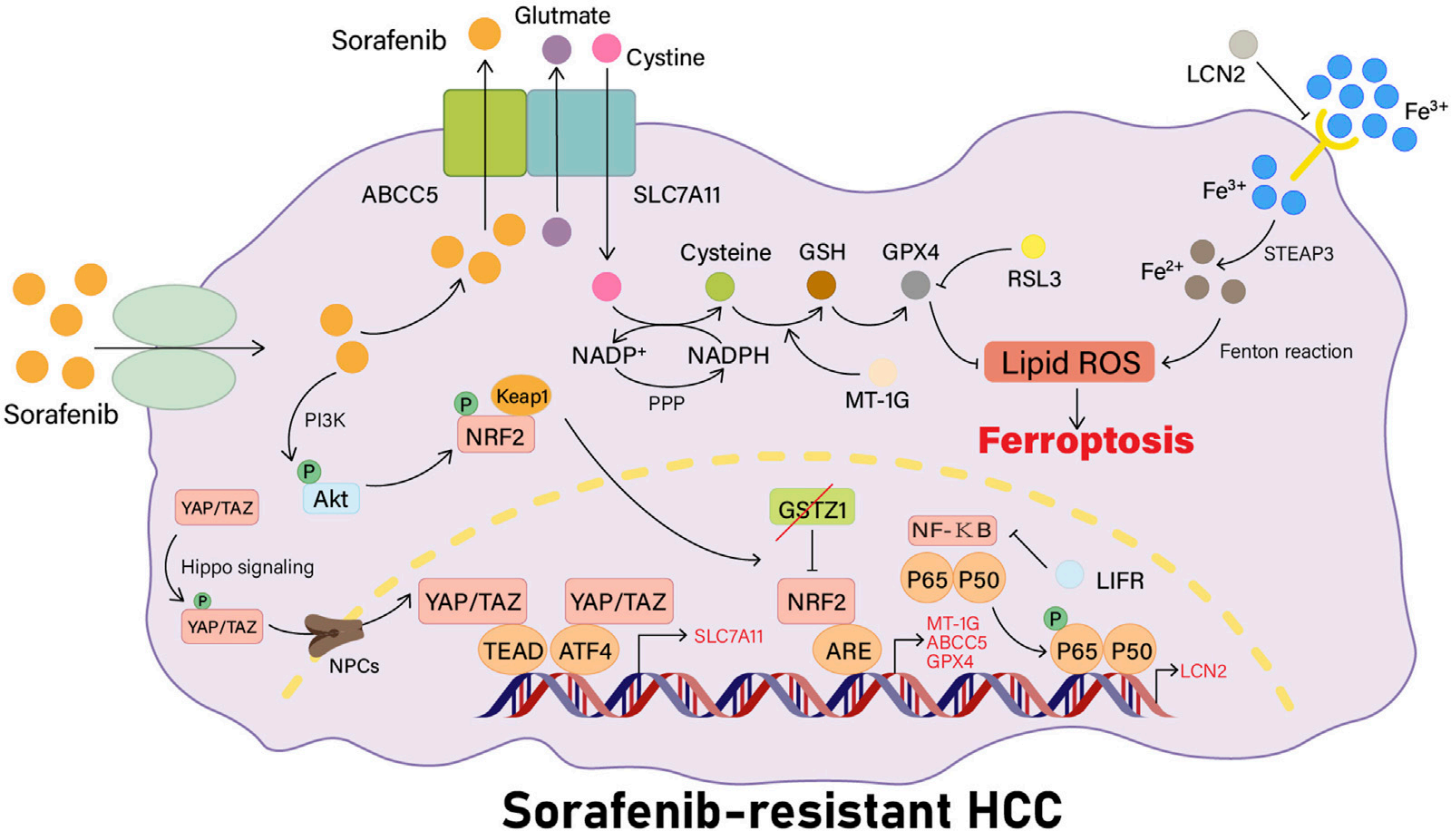
Sorafenib resistance pathways associated with ferroptosis. YAP/TAZ is firstly phosphorylated by Hippo signaling, then transported into the nucleus by NPCs. YAP/TAZ can activate ATF4 in the nucleus or bind to TEAD to induce the expression of SLC7A11 and raise intracellular GSH levels. LCN2 can inhibit the activity of the transferrin receptor, and the deletion of LIFR will activate the NF-κB signaling pathway, leading to the upregulation of LCN2 expression, thus reducing the intracellular iron entrance and inhibiting the occurrence of ferroptosis in cells. When the Keap1-Nrf2 system is activated, the expression of its downstream genes related to ferroptosis, such as MT-1G, ABCC5, and GPX4, will be increased. MT-1G can prevent the lipid peroxidation process in HCC cells. ABCC5 can stabilize the SLC7A11 protein for a more stable action.

YAP/TAZ has recently been shown to be a new regulator of the solute carrier family 7 member 11 (SLC7A11) gene expression. In sorafenib-responsive HCC cells, YAP/TAZ and ATF4 cannot be activated and are not localized to the nucleus, thus they do not activate the expression of SLC7A11 or increase intracellular glutathione levels. In sorafenib-resistant HCC cells, YAP/TAZ and ATF4 are activated in the nucleus, and YAP or TAZ binds to the DNA fragment containing the TEAD motif in the promoter of the SLC7A11 gene to induce the expression of SLC7A11, increasing the intracellular levels of GSH, decreasing the levels of ROS, and inhibiting ferroptosis in HCC cells, which also means that the HCC cells become resistant to sorafenib treatment. In a recent investigation, researchers used immunohistochemistry to investigate the expression and localization of YAP, showing that both the total YAP staining and nuclear YAP staining were more prevalent in HCC tissues than in non-tumorous regions (Qin et al., 2021). In addition, YAP/TAZ maintain ATF4 protein stability, nuclear localization, and transcriptional activity, which in turn cooperatively induces SLC7A11 protein expression (Figure 1) (Gao et al., 2021).

#### 2.2.2 LIFR-NF-κB-LCN2 pathway

The leukemia inhibitory factor (LIF) is the most pleiotropic member of the interleukin-6 family of cytokines (Nicola and Babon, 2015), with a broad spectrum of activity. LIF receptors (LIFRs) are found in various organs and tissues and are expressed in both fetal and adult parenchymal hepatocytes (Hilton, Nicola, and Metcalf, 1991). Lipid transportation protein 2 (LCN2) is an innate immune protein (Xu et al., 2020), which is observed to be highly expressed in a broad range of diseases such as nasopharyngeal carcinoma (Zhang M X et al., 2021), neurodegenerative diseases (Liu R et al., 2022), gliomas (Du et al., 2019), and liver diseases (Asimakopoulou, Weiskirchen, and Weiskirchen, 2016), as well as its interacting proteins. Although LCN2 does not directly associate with iron, it does serve an essential role in iron homeostasis and inflammation by forming ternary complexes with iron transporters as cofactors and thereby interacting with iron (Xiao X et al., 2017). LIFR is a negative regulator of the liver and the LCN2-NF-κB signaling pathway (Yao et al., 2021).

According to recent studies, LIFR expression is reduced in HCC cell lines, and this reduction could be associated with DNA methylation. LIFR deficiency activates the NF-κB signaling pathway through Src homology region 2 (SH2) domain–containing phosphatase 1 (SHP1), causing an increase in LCN2 expression, which results in reduced intracellular iron entry and leads to reduced cellular sensitivity to ferroptosis-inducing agents, thereby promoting liver tumorigenesis. Tumors may be more sensitive to radiation and immunotherapy when LCN2-neutralizing antibodies are applied. High LIFR expression levels and low LCN2 expression levels can be used to estimate the effect of sorafenib, and low expression levels of LIFR and high expression levels of LCN2 can be used to help select patients with HCC who may be benefited from combination treatment with sorafenib and LCN2 neutralizing antibodies (Yao et al., 2021). In conclusion, the present study provides a new direction to target ferroptosis for optimizing HCC cancer treatment strategies (Figure 1).

#### 2.2.3 Keap1-Nrf2 system

Mammals have gradually evolved complex cryoprotective mechanisms throughout evolution to protect cells against oxidative stress and xenobiotics. The Kelch-like ECH-associated protein 1(Keap1)–nuclear factor erythroid 2–related factor 2 (Nrf2) pathway is one of the most crucial mechanisms. Nrf2, a powerful transcriptional activator, plays a pivotal role in regulating the expression of detoxification enzymes and genes encoding antioxidant proteins by combining with antioxidant response elements (AREs)/electrophilic response elements (EpREs) (Morita and Motohashi, 2011). Keap1 is a component of the Cullin 3 (CUL3)–based E3-ubiquitin ligase complex that maintains Nrf2 stability and promotes Nrf2 accumulation (Taguchi and Yamamoto, 2017).

Recent studies have revealed intricate molecular mechanisms of Nrf2 activation responding to stress (Table 1) and have elucidated the link between Nrf2 and numerous human diseases (Morita and Motohashi, 2011). At the same time, recent findings have also suggested that p62-Keap1-NRF2 is a core pathway for ferroptosis inhibition, and the upregulation of MT-1G (Sun et al., 2016a), NQO1, HO-1, FTH1 (Sun et al., 2016b), ABCC5 (Huang et al., 2021), and other NRF2 target genes that are downstream of this pathway can cause resistance to sorafenib by inhibiting the development of intracellular ferroptosis.

**TABLE 1:** Molecules and proteins associated with Nrf2 activation.

Molecules/proteins	Feedback mechanism	Major effects	Reference
GSTZ1	Negative	Inhibits the activation of the Nrf2 pathway	Wang Q et al. (2021)
QSOX1	Negative	Restrains EGF-induced EGFR activation, leading to the suppression of Nrf2 activity	Sun et al. (2021)
SLC27A5	Negative	Inhibits intracellular PUFA lipids and ROS levels and inhibits the activation of the Keap1-Nrf2 pathway	Gao et al. (2020)
FACT complex	Positive	Accelerates the transcription elongation of Nrf2 and its downstream antioxidant genes	Shen et al. (2020)
FNDC5	Positive	Promotes the activation of the PI3K/Akt pathway and increases the level of Nrf2 in the nucleus	Liu H et al. (2022)
SIRT6	Positive	Enhances the Keap1-Nrf2 signaling pathway	Cai et al. (2021)

##### 2.2.3.1 NRF2-MT-1G

Metallothioneins (MTs) are a family of low-molecular-weight proteins with a high cysteine level (15–30%), which provides them with optimal metal coordinating capacity (Atrian and Capdevila, 2013). MTs have a key function in heavy metal detoxification and antioxidant activities (Coyle et al., 2002). There are four main members of mammalian MT: MT-1, MT-2, MT-3, and MT-4 (Dai et al., 2021). MT-1 and MT-2 are broadly expressed in mammals (Coyle et al., 2002). MT-2, MT-3, and MT-4 proteins are encoded by a single gene, whereas MT-1 protein is made up of many isoforms encoded by 13 MT-1 genes, and the known MT-1 active genes are MT-1A, MT-1B, MT-1E, MT-1F, MT-1G, MT-1H, MT-1M, and MT-1X (Thirumoorthy, 2007).

Expression of MT-1G but no other MT types are specifically upregulated in HCC cells in the presence of sorafenib, a tyrosine kinase inhibitor. MT-1G upregulation is mediated by transcription factor Nrf2 (but not p53 or HIF1α) (Sun et al., 2016a). Nrf2 is a significant regulator of the cellular antioxidant response, commanding the expression of genes related to antioxidant and pro-electrical stress, thereby neutralizing ROS to restore the cellular redox balance (Xue, Zhou, and Qiu, 2020). Nrf2 plays a pivotal role in protecting HCC cells against ferroptosis (Sun et al., 2016b). Upregulated MT-1G can cause resistance to sorafenib by inhibiting lipid peroxidation in HCC cells without changing the levels of intracellular iron. Blocking MT-1G expression increases sorafenib’s antitumor activity by inducing ferroptosis both *in vivo* and *in vitro* (Sun et al., 2016a). Sorafenib-induced ferroptosis is inhibited not only by MT-1G but also by the upregulation of other NRF2 target genes such as quinone oxidoreductase-1 (NQO1), heme oxygenase-1 (HO1), and ferritin heavy chain 1 (FTH1) (Figure 1) (Sun et al., 2016b).

##### 2.2.3.2 NRF2-ABCC5

ATP-binding cassette (ABC) transporters are a class of membrane proteins that mediate a wide range of transport processes dictated by ATP (Locher, 2016) and have a significant role in transporting substances across membranes. Human ABC proteins can be categorized into seven subfamilies (A–G) on the basis of the arrangement of the molecular structural elements, i.e., the topology of the nucleotide-binding structural domain and transmembrane structural domain (Toyoda et al., 2008). The C subfamily contains 13 members (Xu et al., 2020), of which the ABCC5 gene is upregulated in the expression of various cancers (such as breast, esophageal, head and neck, kidney, liver, and lung cancers) and has been confirmed to be related to cancer progression (Chen et al., 2021). An investigation in 2015 has shown that ABCC5 may affect the disposition of endogenous metabolites, toxins, and drugs (Jansen et al., 2015).

A recent study using HuH-7, HepG2, and SK-Hep-1 cell lines has revealed that long-term administration of sorafenib activates the PI3K/AKT/Nrf2 pathway in HCC, which is necessary for the induction of ABCC5 expression by sorafenib. Ferroptosis is downregulated by high-expressing ABCC5 through the stabilization of SLC7A11 protein and reduction of GPX4 depletion, inhibition of lipid peroxidation, and increased mitochondrial membrane potential (MMP), which contributes to the development of sorafenib resistance in HCC cells. At the same time, it has been shown that blockade of ABCC5 expression could significantly enhance the antitumor activity of sorafenib *in vivo* by inducing the development of ferroptosis *in vitro* and *in vivo* (Huang et al., 2021). Thus, modulating ABCC5 expression and inducing iron death is a promising therapy for overcoming acquired resistance to sorafenib in HCC cells (Figure 1).

##### 2.2.3.3 GSTZ1-NRF2-GPX4 axis

Glutathione S-transferase Zeta 1 (GSTZ1), commonly known as the maleylacetoacetate isomerase (MAAI), is a glutathione s-transferase (GST) superfamily member (Guo and Zhou, 2019). A deficiency of GSTZ1 induces oxidative stress, resulting in the activation of the Keap1/Nrf2/GPX4 signaling pathway and the promotion of HCC progression (Li et al., 2019). In a recent study, researchers have shown that the downregulation of GSTZ1 expression in sorafenib-resistant HCC cells could inhibit sorafenib-induced cell death through activating the Nrf2 pathway, thereby increasing the expression levels of genes related to iron death (such as GPX4, SLC7A11, and FTL), preventing iron accumulation and lipid peroxidation, and reducing ROS levels. At the same time, GSTZ1 re-expression increases the sensitivity of HCC cells to sorafenib treatment, suggesting a negative regulatory role for GSTZ1 in resistance to sorafenib (Wang Q et al., 2021). Therefore, blockade of the Nrf2/GPX4 pathway to strengthen the anticancer effect of sorafenib through the induction of ferroptosis is a potential therapeutic strategy for HCC (Figure 1).

#### 2.2.4 ETS1-miR-23a-3p-ACSL4 axis

In addition to the abovementioned pathways, certain miRNAs also play an important role in the development of resistance to sorafenib in HCC cells. The most significant of these miRNAs is miR-23a-3p. miR-23a-3p is a negative regulator of iron death that targets the 3'-UTRs of acyl-CoA synthetase long-chain family member 4 (ACSL4) downstream, thereby reducing the generation of ROS and decreasing cellular ferroptosis. ETS proto-oncogene 1 (ETS1), a key transcription factor that directly stimulates the transcription of miR-23a-3p under the treatment of sorafenib, was activated following treatment with sorafenib (Lu et al., 2022). The ETS1-miR-23a-3p-ACSL4 pathway plays a role in HCC cell resistance to sorafenib through the regulation of ferroptosis.

## 3 Induction of ferroptosis reverses sorafenib resistance

Tumors continue to evolve during development, often evolving multiple mechanisms such as limiting the synthesis and peroxidation of polyunsaturated fatty acids bound to polar lipids (PUFAs-PL), limiting unstable iron pools, and promoting the upregulation of their iron-death defense system in order to evade iron death, thereby mitigating the killing effect of iron on cancer cells, causing cancer cells to acquire drug resistance, and promoting the continued development of tumors (Lei, Zhuang, and Gan, 2022).

Ferroptosis has also been shown to play an important role in the development of sorafenib resistance in HCCs. To date, investigators have suggested numerous potential mechanisms of iron death that may reverse sorafenib resistance, such as inhibiting branched-chain aminotransferases 2 (BCAT2), CDGSH iron sulfur domain 2 (CISD2), macropinocytosis, and the Keap1-Nrf2 system (Table 2). In addition, nuclear factor-κB (NF-κB), a sequence-specific DNA binding transcription factor, is overexpressed in almost all cancers and can exert various pro-tumorigenic functions (Hoesel and Schmid, 2013). By targeting downstream of NF-κB, such as the LCN2 gene, we can optimize cancer treatment strategies through targeting ferroptosis.

**TABLE 2:** Additional ways to induce ferroptosis and enhance the effect of sorafenib.

Drugs	Target points/events	Major mechanism	References
Sulfasalazine	BCAT2	Impairs the activation of BCAT2 transcription and reduces the intracellular glutamate level	Wang K. et al. (2021)
Amiloride	Macropinocytosis	Inhibits micropinocytosis and reduces cysteine acquisition	Byun et al. (2022)
Metformin	Nrf2	Induces ferroptosis and enhances the antitumor effect of sorafenib in HCC by inhibiting Nrf2-related pathways	Tang et al. (2022)
CISD2 inhibitors	CISD2	Inhibition of CISD2 promotes Beclin1 action, facilitates autophagy, and increases intracellular iron concentration	Li et al. (2021)
Tiliroside	TBK1	Tiliroside is a TBK1 inhibitor and targets TBK1 to induce ferroptosis	Yang et al. (2023)
Orlistat	FASN	Orlistat is a FASN inhibitor and results in the downregulation of SLC7A11 expression and promotes ferroptosis	Li et al. (2023)

### 3.1 Inhibition of BCAT2 expression by combining sulfasalazine with sorafenib

Branched-chain amino acid aminotransferase (BCATs), namely, BCAT1 and BCAT2 (Nong et al., 2022), are transaminases that function on branched-chain amino acids (BCAAs) such as leucine, isoleucine, and valine and regulate their reversible transamination (Papathanassiu et al., 2017). BCAAs are nitrogen suppliers for the production of glutamate and glutamine, and BCATs are key proteins that can catalyze the reversible ammonification of BCAAs to their respective a-ketoacid (BCKAs) and glutamate (Wang K et al., 2021), that is, BCAT2 can drive the resynthesis of glutamate. Furthermore, the bidirectional transport function of the xCT system in ferroptosis is regulated by the levels of glutamate, and high intracellular glutamate levels increase the activity of the xCT system and promote cystine uptake, thereby inhibiting ferroptosis.

**FIGURE 2:**
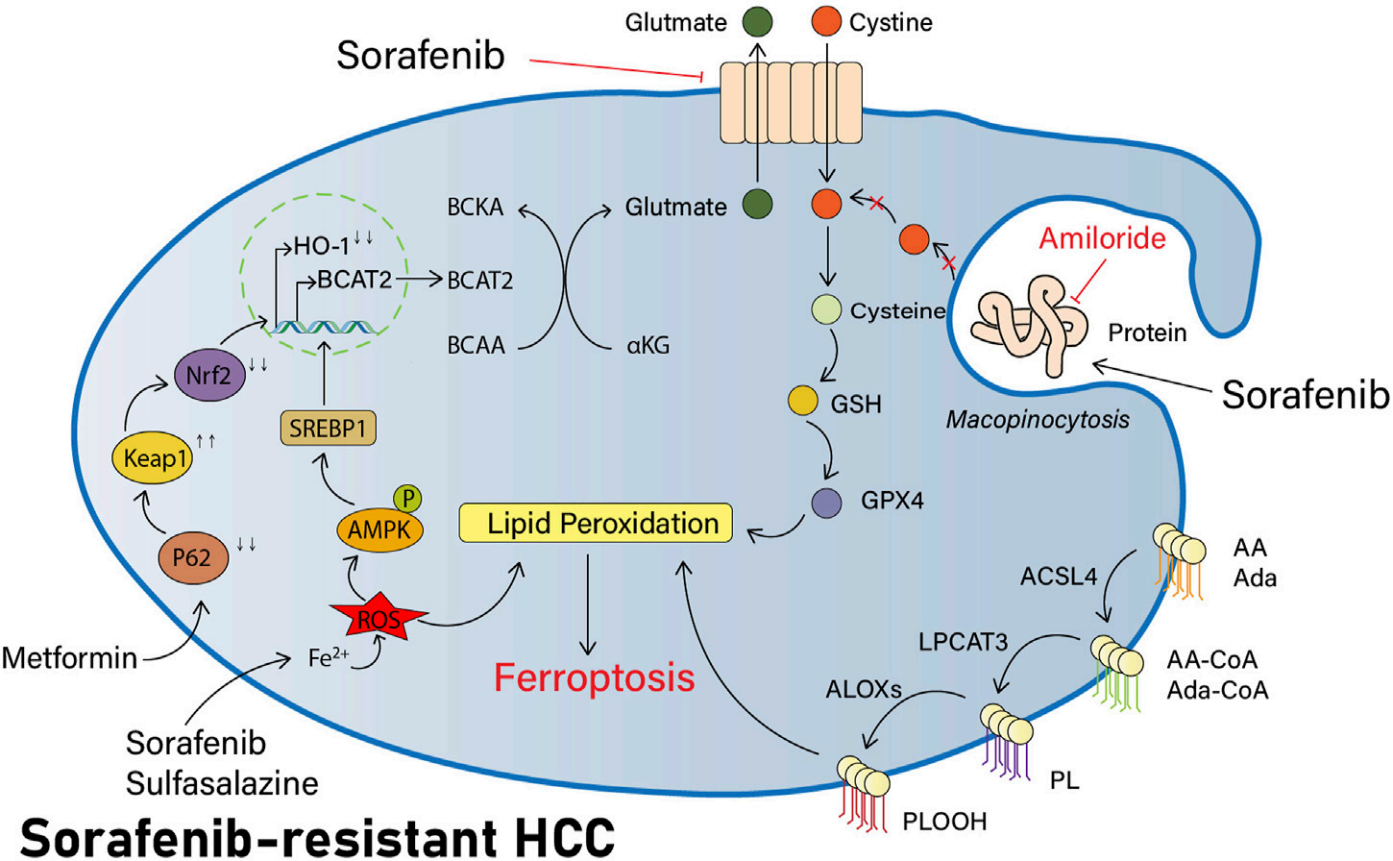
Part of potential pathways to reverse sorafenib resistance by inducing ferroptosis. Sorafenib has synergistic effects with sulfasalazine in that they both induce AMPK phosphorylation of T172, reduce the expression of transcription factor SREBP1, block BCAT2 transcription in the nucleus, reduce intracellular Glu synthesis, and decrease the xCT system activity. NHE inhibitors like amiloride can inhibit macropinocytosis, block the extra acquisition of cysteine by HCC cells, reduce intracellular cysteine levels, and increase lipid peroxidation production. In addition, metformin also modulates the p62-Keap1-NRF2 pathway and decreases HO-1 expression, thereby regulating the ROS response and inducing more ferroptosis in HCC cells.

Previous investigators have demonstrated in pancreatic ductal adenocarcinoma (PDAC) studies that AMP-activated protein kinase (AMPK) can inhibit the nuclear translocation of sterol response element binding protein 1 (SREBP1), thus repressing transcription of its direct BCAT2 target gene (Dey et al., 2017). More recently, investigators have also performed this study in HCC cells. The knockdown of SREBP1 in HCC cells has revealed that BCAT2 expression is also significantly reduced, thereby confirming that the level of BCAT2 expression is also regulated via the AMPK-SREBP1 signaling pathway in HCC cells. Sulfasalazine is an anti-inflammatory medication. Both sorafenib and sulfasalazine can induce the phosphorylation of AMPK on threonine residue 172 (T172) and reduce the expression of transcription factor SREBP1, which further impairs its ability to activate BCAT2 transcription in the nucleus. As a result, the investigators speculate that sorafenib and sulfasalazine might have a collaborative effect in the induction of ferroptosis. Investigators have measured intracellular BCAT2 mRNA and protein expression levels following their combined use and found that both had been significantly reduced in expression. Concurrently, greater tumor shrinkage was seen in a murine model of HCC with the combination of both drugs when comparing their distinct usage, together confirming the plausibility of this conjecture (Wang K et al., 2021) and providing a novel option with great promise for research to improve the efficacy of sorafenib (Figure 2).

### 3.2 Inhibition of CISD2 and restoration of sorafenib-induced ferroptosis

NEET belongs to a recently discovered highly conservative group of iron-sulfur (2Fe-2S) proteins (Nechushtai et al., 2020). It is highly expressed in a variety of cancers, supports cell proliferation and tumor growth, and enhances metastasis (Mittler et al., 2019). A recent work has revealed that the NEET protein cluster plays an important role in the regulation of iron, calcium, and ROS homeostasis in cancer cells (Tamir et al., 2015). We observed three NEET proteins in human cells: CISD1 protein (situated in the outer mitochondrial membrane), CISD2 (situated in the outer mitochondrial membrane, the endoplasmic reticulum, and the membrane connecting the mitochondria to the endoplasmic reticulum), and CISD3 (situated in the mitochondria) (Wang et al., 2020). The most recent study identified CISD2 as a new biomarker for the first time for the detection of ferroptosis-associated drug resistance generated in HCC cells induced by sorafenib and regulated by CISD2 via autophagy (Li et al., 2021).

Autophagy is a highly conserved cellular degradation and recycling process (Parzych and Klionsky, 2014). Indeed, it is currently well recognized that autophagy plays a dynamic dual role in both early tumorigenesis and subsequent tumor formation (Li J et al., 2020). In addition, in the early stages of tumorigenesis, autophagy may control tumor cell proliferation (White, 2012) and may inhibit angiogenesis, thus exerting anticancer effects. Alternatively, when tumors progress into the advanced stages, autophagy may enhance stress tolerance of cancer cells and contribute to their better survival, as well as to their resistance to therapy (Wu et al., 2012). However, excessive autophagy activation may promote iron ion release by degrading ferritin (Hou et al., 2016), leading to an increase in unstable iron, which induces oxidative stress (Carocci et al., 2018) and has a significant role in the induction of ferroptosis. The cytoprotective properties of autophagy have already aroused the interest of clinicians (Dikic and Elazar, 2018), leading to more extensive attention to clinical adjuvant therapy targeting autophagy.

Beclin1, the first discovered mammalian autophagy protein, has been shown to play an active function in regulating autophagy and apoptosis, as a nexus point between autophagy, endosomal, and cell death pathways (Funderburk, Wang, and Yue, 2010). Beclin1 is expressed in many human and mouse tissues, and its expression is primarily located in cytoplasmic structures such as in the endoplasmic reticulum and mitochondria, around the nuclear membrane, and localized to the cytoplasm (Kang et al., 2011). CISD2 binds Beclin1 in the endoplasmic reticulum, thus inhibiting the initiation of autophagy (Sun, Meng, and Wang, 2017).

**FIGURE 3:**
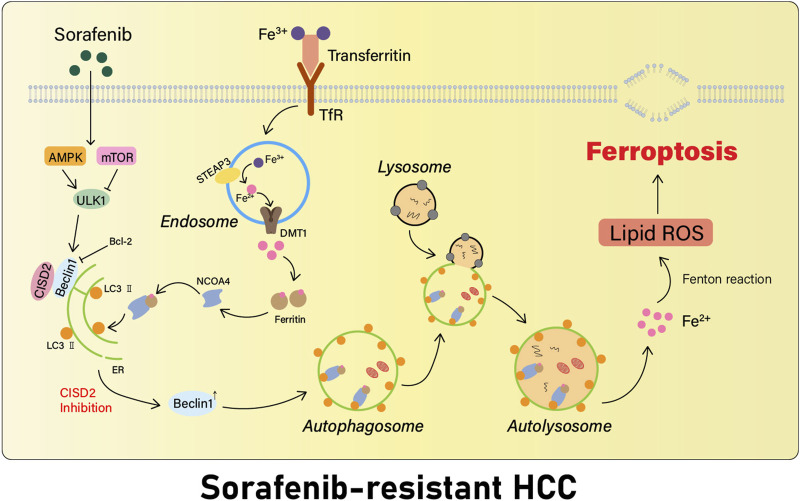
Inhibition of CISD2 restores sorafenib-induced ferroptosis. Inhibition of CISD2 promotes the action of the autophagy regulator Beclin 1, which facilitates the initiation of autophagy in sorafenib-resistant HCC cells, leading to an increase in the intracellular iron content, provoking Fenton response, inducing oxidative stress, and triggering the development of more ferroptosis.

In summary, the inhibition of CISD2 promotes Beclin1 action, facilitates autophagy that occurs in HCC cells resistant to sorafenib, and increases the intracellular iron concentration (Li et al., 2021) (Figure 3). Beclin1 can also play a significant role in promoting ferroptosis development by combining to SLC7A11 and blocking the activity of the xCT system in a direct way (Song et al., 2018), leading to a decrease in the generation of intracellular Glu. Investigations in CISD2 offer a new potential therapeutic approach for the targeted treatment of drug-resistant cancer.

### 3.3 Inhibition of macropinocytosis by using amiloride

Macropinocytosis is an endocytic process driven by actin cytoskeleton (Kay, 2021) and not dependent on network proteins. Macropinocytosis provides an uptake of liquids, particles, and extracellular proteins, which is non-selective and unregulated by its transporters, unlike the phagocytic pathway (Xiao F et al., 2017). Recent studies on the metabolism of cancer have shown that some transformed cells use macropinocytosis to ingest and digest macromolecules in a non-selective manner, enabling them to be used as fuel to support cancer cell survival and proliferation, thereby supporting their growth in a nutrient-poor tumor environment (Palm, 2019). Additional studies have confirmed that macropinocytosis is induced by sorafenib in human HCC tissues and hepatocytes. Macropinocytosis may provide a novel route of cysteine acquisition for HCC cells, thereby reducing sorafenib-induced ferroptosis and decreasing HCC cells’ sensitivity to sorafenib (Byun et al., 2022).

Macropinocytosis is distinct from other types of endocytosis in that it is uniquely sensitive to inhibitors of Na^+^/H^+^ exchange (NHE) (Mishra et al., 2022). Amiloride, a potassium-preserving diuretic, is a clinically used NHE antagonist that inhibits Na^+^ reabsorption mediated by Na^+^/H^+^ exchangers in the epithelium of the distal convoluted tubule of the kidney and the collecting duct (Teiwes and Toto, 2007). Experimental studies have shown that amiloride can perturb the Na^+^/H^+^ exchanger, preventing the burst of increased metabolic acid produced by intracellular metabolic pathways from being carried out of the cell, thereby decreasing the intracellular pH and inhibiting the activation of the RAC1 and CDC42 signaling pathways, which is a key step in the generation of macropinocytosis in a pH-dependent manner (Koivusalo et al., 2010).

In other words, the use of NHE inhibitors such as amiloride inhibits macropinocytosis, therefore improving the sensitivity of HCC cells to sorafenib-induced ferroptosis (Figure 2).

### 3.4 Inhibition of Nrf2-associated pathways by using metformin

As mentioned previously, the Keap1-Nrf2 system is one of the important pathways by which ferroptosis occurs in HCC cells and is involved in the proliferation and chemoresistance of HCC cells. The current studies have demonstrated that metformin can induce ferroptosis and enhance the antitumor effect of sorafenib in HCC by inhibiting Nrf2-related pathways (Tang et al., 2022).

Metformin is an antidiabetic drug of the biguanide group and is a first-line drug for the therapy of type 2 diabetes and has valuable research implications for treating cardiovascular disease and cancer (Foretz et al., 2014). The use of metformin has been shown in population-based studies to help reduce the risk of cancer and improve prognosis (Pollak, 2012).

Metformin may interfere with iron delivery to cells (Stynen et al., 2018) and disrupt intracellular iron homeostasis, and it is hypothesized that it may have some relationship to ferroptosis. Our previous research studies have demonstrated that in breast cancer cells, metformin is capable of inducing ferroptosis through the inhibition of UFMylation of SLC7A11, a ubiquitin-like modification that has a significant effect on the development and progression of breast cancer, thus negatively regulating the stability of the SLC7A11 protein and ultimately inducing ferroptosis (Yang et al., 2021). Similarly, in their exploration of HCC, the investigators found that metformin was capable of inducing ferroptosis in HCC cells. HO-1 is the main protein regulating the ROS response and is the downstream of Nrf2. Metformin can inhibit the translocation of Nrf2, thus reducing HO-1 expression and inducing further ferroptosis, and significantly inhibit the colonization of HCC cells *in vitro* and *in vivo* (Figure 2) (Tang et al., 2022).

In patients who fail apoptosis and necrosis induction therapy, additional ferroptosis may still be beneficial if it can be induced at all. This implies that the combined use of metformin and sorafenib induces ferroptosis in HCC cells that are already resistant to sorafenib and improves the cytotoxicity of sorafenib on HCC cells, thereby inhibiting the growth of HCC tumors.

## 4 Conclusion and opinions

Ferroptosis research in cancer has expanded rapidly in recent years, which is considered to play a significant role in anticancer therapies (Zhang et al., 2022). Among the malignancies, primary liver cancer has a high incidence and high mortality rate. Also, HCC is one of the most common types, accounting for as much as 90% (Chen et al., 2015), with a poor prognosis. For patients with unresectable advanced HCC, sorafenib chemotherapy is currently the preferred treatment (Zhang W et al., 2021).

Sorafenib can promote ferroptosis in HCC cells through a dual mode of action: one is retinoblastoma (Rb) protein independent, which is associated with its ability to deplete GSH in HCC cells. The other depends on the Rb protein status of HCC cells and is associated with increased mitochondrial production of ROS (Louandre et al., 2015). Recent studies have demonstrated that additional induction of intracellular ferroptosis can significantly improve the noted efficacy of sorafenib, particularly in human HCC cells that have already acquired chemotherapy resistance to sorafenib. Using multikinase inhibitors such as lenvatinib in patients with HCC can silence FGFR4, thereby inhibiting the expression of the xCT system, inducing the accumulation of ROS, and causing ferroptosis in HCC cells (Iseda et al., 2022). The use of metformin and other drugs can inhibit Nrf2-related pathways, induce ferroptosis, and effectively enhance the sensitivity of HCC cells to ferroptosis (Tang et al., 2022). This effect can also be obtained by using orlistat, a pharmacological inhibitor of fatty acid synthase (FASN) (Li et al., 2023), and by using tiliroside, an inhibitor of TANK-binding kinase 1 (TBK1) (Yang et al., 2023). In addition, various cell death pathways or transmembrane transport pathways such as autophagy (Li et al., 2021) and macropinocytosis (Byun et al., 2022) also have a significant function in the development of ferroptosis induction, providing several potential avenues of research to reverse sorafenib resistance.

In summary, ferroptosis induction in HCC cells using the methods outlined above has emerged as one of the potential avenues of research for the reversal of resistance to sorafenib and has offered a trustworthy basis for the exploration of novel therapies for HCC. In addition, gradual further research in the fields of epigenetics, tumor microenvironment, and immune checkpoints has also provided more directions to explore the principles of sorafenib resistance. At the same time, there are still many questions to be explored, such as whether the overall survival of HCC patients can be substantially increased after the reversal of sorafenib resistance by different methods. We believe that soon the scientific and rational combination of drugs to improve the efficacy of chemotherapy regimens will become a reality, and investigators will seek novel strategies for treating HCC and other malignancies based on the ferroptosis theory.

## Abbreviations

BCAT2, branched-chain aminotransferases 2; CISD2, CDGSH iron sulfur domain 2; EGFR, epidermal growth factor receptor; FACT, facilitates chromatin transcription; FASN, fatty acid synthase; FNDC5, fibronectin type III domain-containing 5; GSTZ1, glutathione S-transferase Zeta 1; Keap1, Kelch-like ECH-associated protein 1; Nrf2, nuclear factor E2-related factor 2; PI3K, phosphatidylinositol-3-kinase; PUFA, polyunsaturated fatty acid; QSOX1, quiescin sulfhydryl oxidase 1; ROS, reactive oxygen species; SIRT6, sirtuin 6; SLC27A5 gene, solute carrier family 27 member 5 gene; and TBK1, TANK-binding kinase 1.
