# Supplementary material for: Mechanism of sorafenib resistance associated with ferroptosis in HCC
Source: Front Pharmacol. 2023 Jun 7;14:1207496. doi: 10.3389/fphar.2023.1207496 (PMC10282186; doi:10.3389/fphar.2023.1207496)

Supplementary Material

**Mechanism of Sorafenib Resistance Associated with Ferroptosis in HCC**

**Lingling Guo^1^; Cuntao Hu^1^; Mengwen Yao^1^; Guang Han^1*^**

*** Correspondence:** Corresponding Author: cmu_hg@163.com

## Supplementary Figures


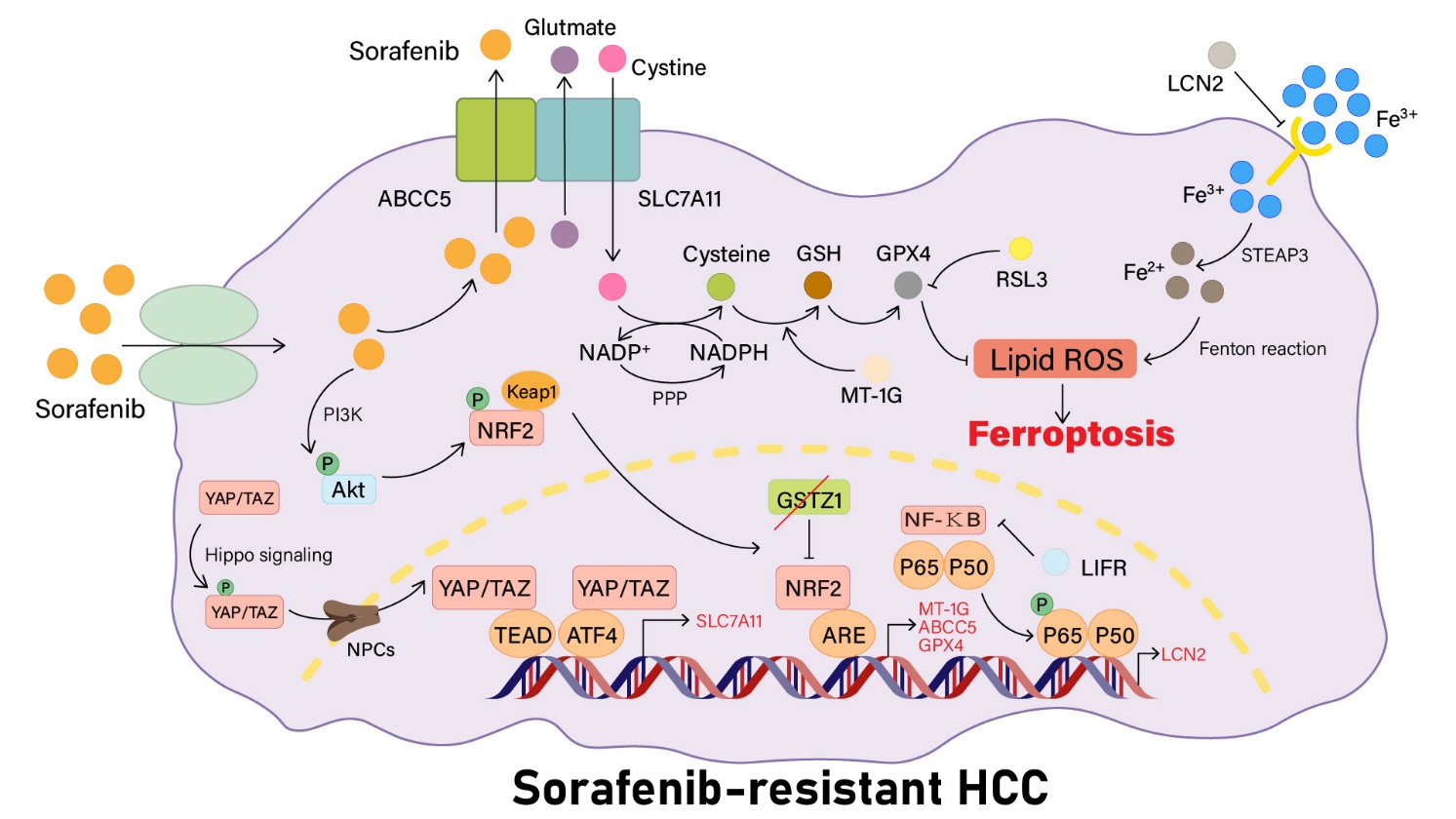


**Supplementary Figure 1.** **Sorafenib Resistance Pathways Associated with Ferroptosis.**

YAP/TAZ is firstly phosphorylated by Hippo signaling, then transported into the nucleus by NPCs. YAP/TAZ can activate ATF4 in the nucleus or bind to TEAD to induce the expression of SLC7A11 and raise intracellular GSH levels. LCN2 can inhibit the activity of the transferrin receptor, and the deletion of LIFR will activate the NF-κB signaling pathway, leading to the upregulation of LCN2 expression, thus reducing the intracellular iron entrance and inhibiting the occurrence of ferroptosis in cells. When the Keap1-Nrf2 system is activated, the expression of its downstream genes related to ferroptosis, such as MT-1G, ABCC5, and GPX4, will be increased. MT-1G can prevent the lipid peroxidation process in HCC cells. ABCC5 can stabilize the SLC7A11 protein for a more stable action.


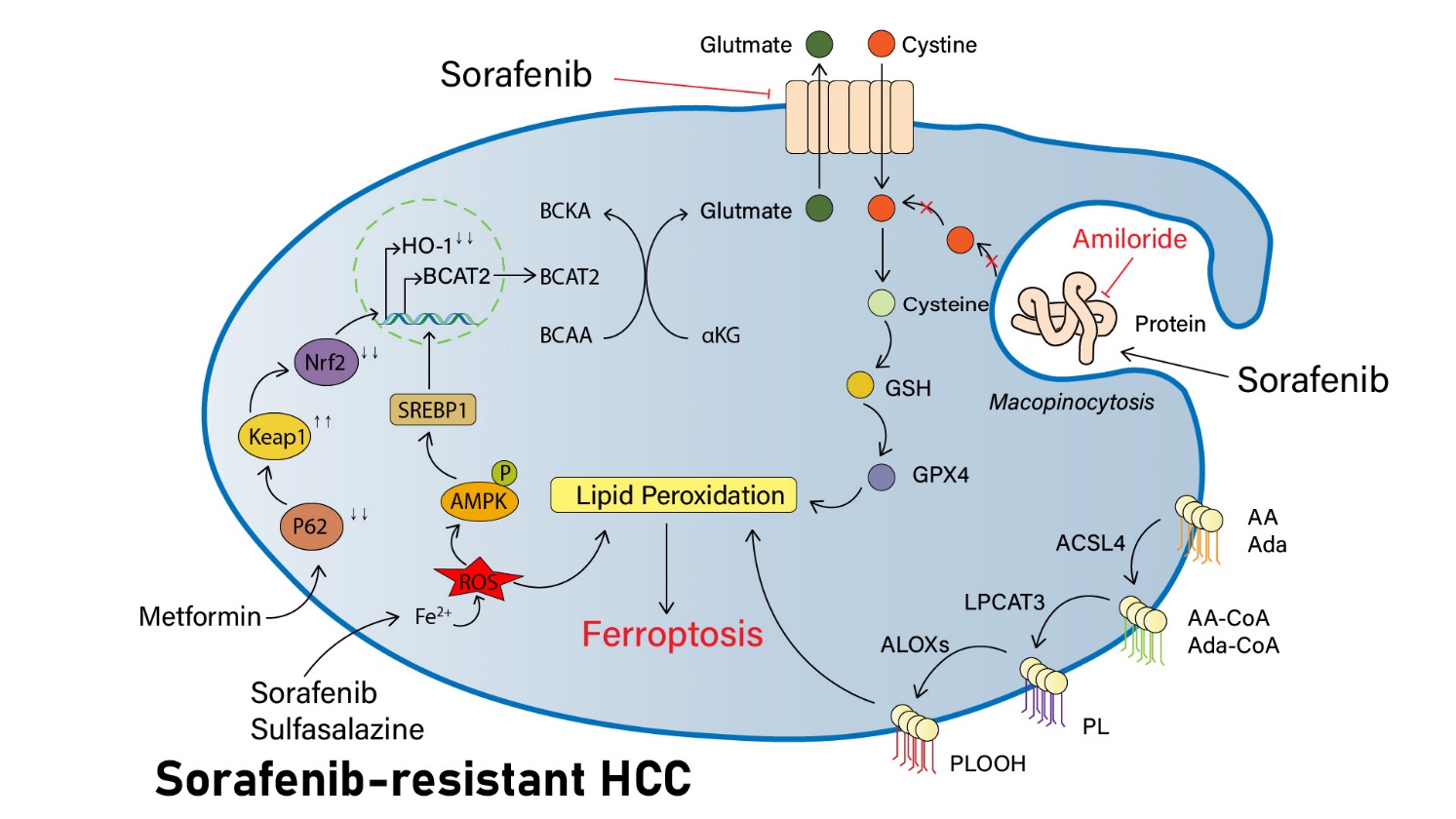


**Supplementary** **Figure 2.** **Part of potential pathways to reverse Sorafenib resistance by inducing Ferroptosis.**

Sorafenib has synergistic effects with sulfasalazine in that they both induce AMPK phosphorylation of T172, reduce the expression of transcription factor SREBP1, block BCAT2 transcription in the nucleus, reduce intracellular Glu synthesis, and decrease xCT system activity. NHE inhibitors like amiloride can inhibit macropinocytosis, block the extra acquisition of cysteine by HCC cells, reduce intracellular cysteine levels, and increase lipid peroxidation production. In addition, metformin also modulates the p62-Keap1-NRF2 pathway and decreases HO-1 expression, thereby regulating the ROS response and inducing more ferroptosis in HCC cells.


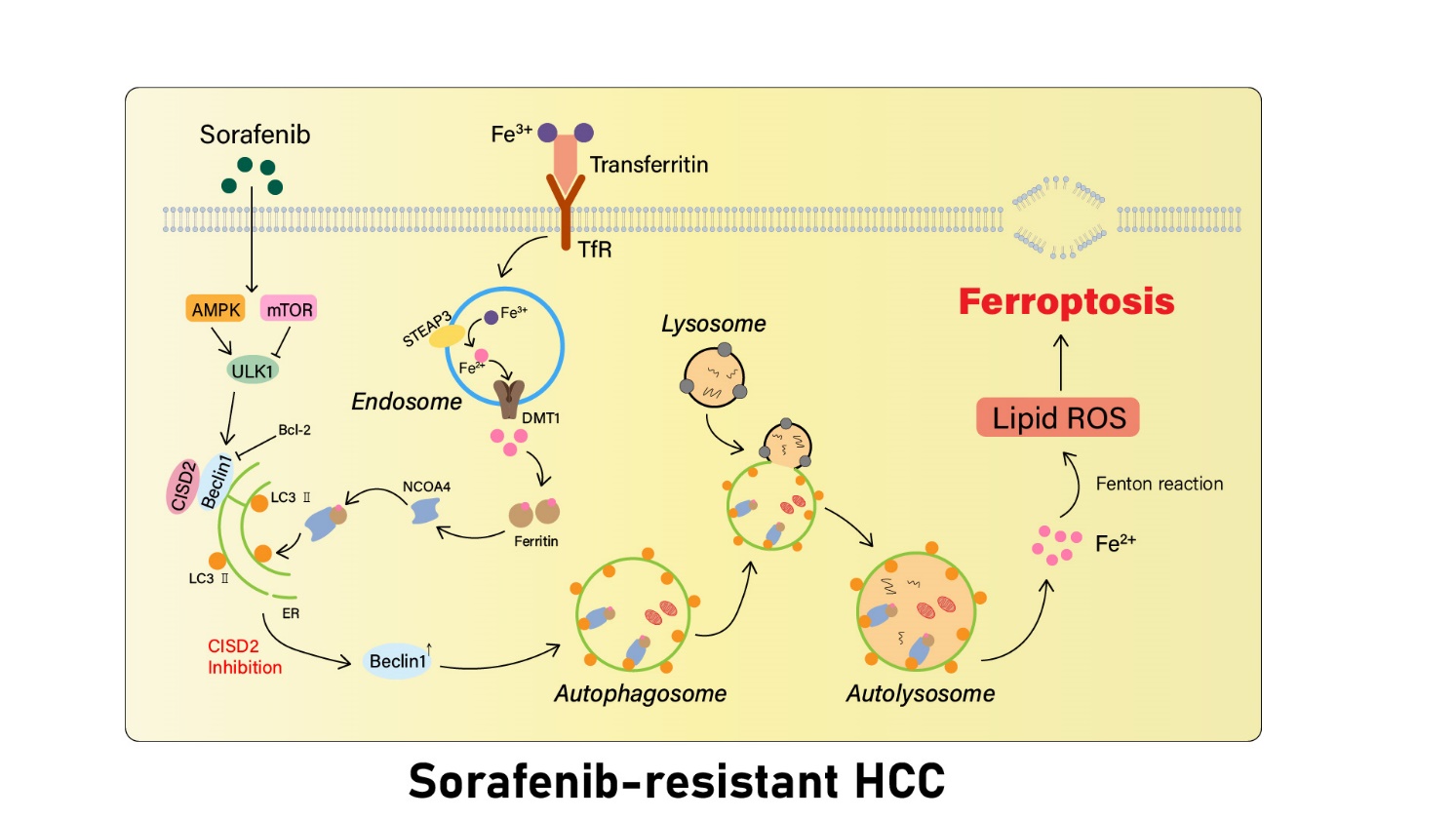


**Supplementary Figure 3.** **Inhibition of CISD2 restores sorafenib-induced ferroptosis.**

Inhibition of CISD2 promotes the action of the autophagy regulator Beclin 1, which facilitates the initiation of autophagy in sorafenib-resistant HCC cells, leading to an increase in intracellular iron content, provoking Fenton response, inducing oxidative stress and triggering the development of more ferroptosis.

## Supplementary Tables

| **Molecules/proteins** | **Feedback mechanism** | | **Major effects** | **References** |
| --- | --- | --- | --- | --- |
| GSTZ1 | | negative | inhibit the activation of the Nrf2 pathway | (Q. Wang et al. 2021) |
| QSOX1 | | negative | restrain EGF-induced EGFR activation, lead to suppression of Nrf2 activity | (J. Sun et al. 2021) |
| SLC27A5 | | negative | inhibit intracellular PUFA lipids and ROS levels, inhibit the activation of the Keap1-Nrf2 pathway | (Q. Gao et al. 2020) |
| FACT complex | | positive | accelerate the transcription elongation of Nrf2 and its downstream antioxidant genes | (Shen et al. 2020) |
| FNDC5 | | positive | promote the activation of the PI3K/Akt pathway, increase the level of Nrf2 in the nucleus | (H. Liu et al. 2022) |
| SIRT6 | | positive | enhance the Keap1-Nrf2 signaling pathway | (Cai et al. 2021) |

**Supplementary Table 1. Molecules associated with Nrf2 activation.**

**Abbreviations:**

GSTZ1: glutathione S-transferase zeta 1,

QSOX1: quiescin sulfhydryl oxidase 1,

SLC27A5: solute carrier family 27 member 5 gene,

FACT: facilitates chromatin transcription,

FNDC5: fibronectin type III domain containing 5,

SIRT6: sirtuins 6,

Nrf2: nuclear factor E2-related factor 2,

EGFR: epidermal growth factor receptor,

PUFA: polyunsaturated fatty acid,

ROS: reactive oxygen species,

Keap1: kelch-like ECH-associated protein 1,

PI3K: phosphatidylinositol-3-kinase

| **Drugs** | **Target points/events** | **Major mechanism** | **References** |
| --- | --- | --- | --- |
| Sulfasalazine | BCAT2 | Sulfasalazine impairs the activation of BCAT2 transcription and reduces the glutamate level intracellular. | (K. Wang et al. 2021) |
| Amiloride | Macropinocytosis | Amiloride inhibits micropinocytosis and reduces cysteine acquisition. | (Byun et al. 2022) |
| Metformin | Nrf2 | Metformin can induce ferroptosis and enhance the anti-tumor effect of sorafenib in HCC by inhibiting Nrf2-related pathways. | (Tang et al. 2022) |
| CISD2 inhibitors | CISD2 | Inhibition of CISD2 promotes Beclin1 action, facilitates autophagy, and increases intracellular iron concentration. | (B. Li et al. 2021) |
| Tiliroside | TBK1 | Tiliroside is a TBK1 inhibitor, which targets TBK1 to induce ferroptosis. | (C. Yang et al. 2023) |
| Orlistat | FASN | Orlistat, a FASN inhibitor, results in the downregulation of SLC7A11 expression and promotes ferroptosis. | (Y. Li et al. 2023) |

**Table 2. Additional ways to induce ferroptosis and enhance the effect of sorafenib.**

**Abbreviations:**

BCAT2: branched-chain aminotransferases 2

CISD2: CDGSH iron sulfur domain 2

TBK1: TANK-binding kinase 1

FASN: fatty acid synthase

## Graphical abstract


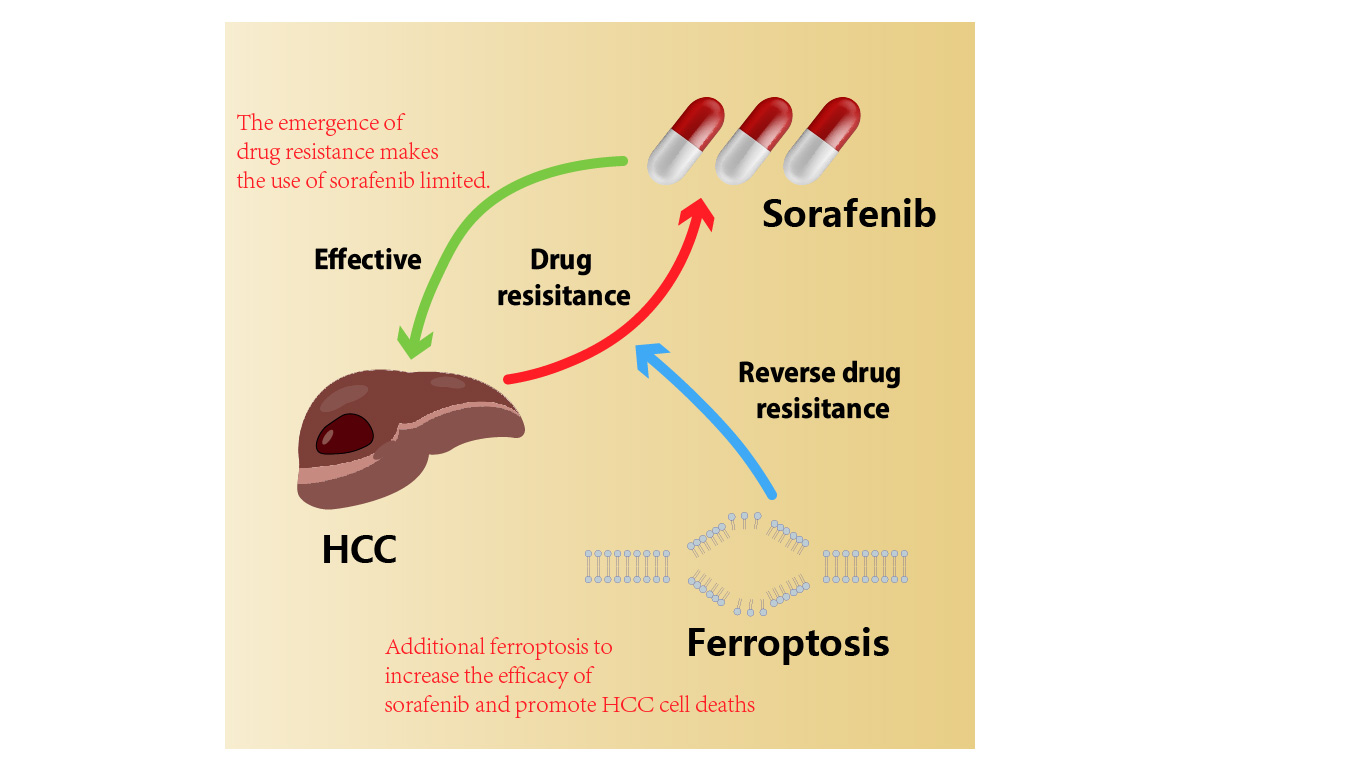

Supplement: Supplementary file 1 [file DataSheet1.docx]
